# Supplementary material for: Multimodal principal component analysis to identify major features of white matter structure and links to reading
Source: PLoS One. 2020 Aug 14;15(8):e0233244. doi: 10.1371/journal.pone.0233244 (PMC7428127; doi:10.1371/journal.pone.0233244)
Supplement: S1 Table — No correlations remained significant after correction for multiple comparisons. (DOCX) [file pone.0233244.s001.docx]

**S1 Table. Correlations between measures included in the final PCA model and Total Reading in all investigated regions. No correlations remained significant after correction for multiple comparisons.**

| Region |  | FA | MD | AD | RD | NDI | ODI | MTR | qihMT | VF_m_ | g-ratio |
| --- | --- | --- | --- | --- | --- | --- | --- | --- | --- | --- | --- |
| Left arcuate | r | -0.130 | -0.028 | -0.121 | 0.031 | 0.175 | -0.008 | 0.195 | 0.313 | -0.005 | 0.041 |
|  | p | 0.399 | 0.856 | 0.435 | 0.844 | 0.256 | 0.959 | 0.205 | 0.039 | 0.976 | 0.792 |
| Right arcuate | r | -0.111 | -0.035 | -0.121 | 0.018 | 0.118 | -0.079 | 0.146 | 0.378 | 0.100 | -0.081 |
|  | p | 0.479 | 0.825 | 0.440 | 0.909 | 0.452 | 0.614 | 0.349 | 0.012 | 0.524 | 0.606 |
| Left ILF | r | -0.093 | 0.169 | 0.135 | 0.168 | 0.073 | -0.201 | 0.145 | 0.348 | -0.122 | 0.113 |
|  | p | 0.548 | 0.273 | 0.381 | 0.274 | 0.638 | 0.190 | 0.349 | 0.021 | 0.432 | 0.465 |
| Right ILF | r | 0.003 | 0.123 | 0.161 | 0.046 | -0.021 | -0.147 | 0.109 | 0.328 | 0.069 | -0.076 |
|  | p | 0.982 | 0.427 | 0.295 | 0.766 | 0.894 | 0.340 | 0.481 | 0.030 | 0.659 | 0.626 |
| Left IFOF | r | 0.014 | -0.056 | -0.052 | -0.047 | 0.116 | -0.188 | 0.282 | 0.283 | -0.047 | 0.070 |
|  | p | 0.930 | 0.719 | 0.739 | 0.763 | 0.454 | 0.221 | 0.067 | 0.062 | 0.763 | 0.650 |
| Right IFOF | r | -0.099 | -0.056 | -0.107 | -0.026 | 0.053 | -0.132 | 0.167 | 0.373 | 0.028 | -0.017 |
|  | p | 0.521 | 0.716 | 0.488 | 0.867 | 0.732 | 0.394 | 0.279 | 0.013 | 0.855 | 0.914 |
| Left uncinate | r | -0.237 | 0.077 | -0.108 | 0.145 | 0.106 | -0.009 | 0.170 | 0.098 | -0.238 | 0.266 |
|  | p | 0.121 | 0.622 | 0.485 | 0.347 | 0.493 | 0.954 | 0.269 | 0.527 | 0.119 | 0.081 |
| Right uncinate | r | -0.010 | -0.107 | -0.107 | -0.099 | 0.041 | -0.080 | 0.182 | 0.332 | -0.070 | 0.100 |
|  | p | 0.950 | 0.490 | 0.491 | 0.521 | 0.792 | 0.604 | 0.236 | 0.028 | 0.650 | 0.518 |
| Splenium | r | 0.206 | -0.087 | 0.019 | -0.130 | 0.086 | -0.177 | 0.224 | 0.204 | 0.039 | 0.066 |
|  | p | 0.180 | 0.575 | 0.903 | 0.399 | 0.578 | 0.250 | 0.143 | 0.185 | 0.803 | 0.672 |
